# Nutritional and Dietary Management of Chronic Kidney Disease Under Conservative and Preservative Kidney Care Without Dialysis

**DOI:** 10.1053/j.jrn.2023.06.010

**Published:** 2023-06-30

**Authors:** Connie M. Rhee, Angela Yee-Moon Wang, Annabel Biruete, Brandon Kistler, Csaba P. Kovesdy, Diana Zarantonello, Gang Jee Ko, Giorgina Barbara Piccoli, Giacomo Garibotto, Giuliano Brunori, Keiichi Sumida, Kelly Lambert, Linda W. Moore, Seung Hyeok Han, Yoko Narasaki, Kamyar Kalantar-Zadeh

**Affiliations:** * Division of Nephrology, Hypertension and Kidney Transplantation, University of California Irvine, Orange, California; † University Department of Medicine, Queen Mary Hospital, The University of Hong Kong, Hong Kong; ‡ Department of Nutrition Science, Purdue University, West Lafayette, Indiana; § Division of Nephrology, Indiana University School of Medicine, Indianapolis, Indiana; ¶ Division of Nephrology, University of Tennessee Health Science Center, Memphis, Tennessee; ** Nephrology Section, Memphis Veterans Affairs Medical Center, Memphis, Tennessee; †† Nephrology and Dialysis Unit, Azienda Provinciale per i Servizi Sanitari (APSS), Trento, Italy; ‡‡ Department of Internal Medicine, Korea University College of Medicine, Seoul, South Korea; §§ Nephrologie, Centre Hospitalier du Mans, Le Mans, France; ¶¶ Department of Internal Medicine, University of Genoa, Genova, Italy; *** School of Medical, Indigenous and Health Sciences, Faculty of Science, Medicine and Health, University of Wollongong, Wollongong, New South Wales, Australia; ††† Department of Surgery, Houston Methodist Hospital, Houston, Texas; ‡‡‡ Department of Internal Medicine, Yonsei University College of Medicine, Seoul, South Korea; §§§ The Lundquist Institute at Harbor-UCLA Medical Center, Torrance, California; ¶¶¶ Nephrology Section, Tibor Rubin Veterans Affairs Medical Center, Long Beach, California

**Keywords:** Nutrition, conservative management, preservative management, chronic kidney disease, plant-based diets

## Abstract

While dialysis has been the prevailing treatment paradigm for patients with advanced chronic kidney disease (CKD), emphasis on conservative and preservative management in which dietary interventions are a major cornerstone have emerged. Based on high-quality evidence, international guidelines support the utilization of low-protein diets as an intervention to reduce CKD progression and mortality risk, although the precise thresholds (if any) for dietary protein intake vary across recommendations. There is also increasing evidence demonstrating that plant-dominant low-protein diets reduce the risk of developing incident CKD, CKD progression, and its related complications including cardiometabolic disease, metabolic acidosis, mineral and bone disorders, and uremic toxin generation. In this review, we discuss the premise for conservative and preservative dietary interventions, specific dietary approaches used in conservative and preservative care, potential benefits of a plant-dominant low-protein diet, and practical implementation of these nutritional strategies without dialysis.

## Introduction: Scope and International Trends of Advanced Chronic Kidney Disease

GLOBALLY, THERE HAS been growing recognition of the critical importance of conservative and preservative measures to mitigate the incidence and progression of chronic kidney disease (CKD).^1–5^ Nearly four million people in the world have end-stage kidney disease (ESKD) treated with kidney replacement therapy, with the majority (69%) receiving hemodialysis.^6^ With respect to international comparisons, the United States incidence rate (410 per million) trails closely behind that of parts of Mexico (570 and 483 per million in Jalisco and Aguascalientes, respectively) and Taiwan (529 per million) as the geographic catchments with the highest rates of de novo ESKD.^7^

International data also show an aging and ailing incident ESKD population worldwide.^7–9^ Across Western high income countries, age-stratified analyses show that the highest incidence of treated ESKD is among patients ≥75 years of age.^7^ Data from the National Institutes of Health sponsored “Transitions of Care in CKD” United States Renal Data System Special Study have also shown that, among people living with advanced CKD transitioning to ESKD, there is a high burden of comorbidities, including diabetes (74%), heart failure (59%), depression (33%), and anxiety (10%),^10^ as well as symptom burden.^2,11,12^ While dialysis has been the prevailing treatment paradigm among patients with advanced CKD who are ineligible for, or unlikely to, receive preemptive kidney transplantation, there has been growing emphasis on conservative and preservative measures. These approaches include a strong emphasis on dietary interventions, in order to reduce estimated glomerular filtration rate (eGFR) decline, optimize health-related quality of life (HRQOL), stabilize metabolic status,^13^ and mitigate the unpleasant symptoms and complications of uremia.^1–4^ In this review, we discuss 1) the rationale for conservative and preservative dietary management approaches, 2) specific dietary interventions in the conservative and preservative care of advanced CKD, 3) potential benefits of a low-protein and plant-dominant diet, including data on kidney, metabolic, and survival outcomes, and 4) the practical implementation of dietary interventions in conservative and preservative care.

## Revisiting Traditional Paradigms in the Management of Advanced CKD

For approximately 5 decades, dialysis has been the default treatment strategy for ESKD globally. In 1972, the United States Congress approved the 1972 Medicare End-Stage Renal Disease Program which ended rationing of dialysis due to limited resources and led to near-universal access to this form of renal replacement therapy.^14^ However, an unintended consequence of uninhibited access to dialysis has been the limited progress and innovation in alternative dialysis-free treatment strategies of ESKD until recently.^3,4^

Multiple studies show that dialysis, while life-extending, may not always exert the intended effect of restoring health nor improving outcomes in patients with advanced nondialysis dependent (NDD) CKD who are transitioning to ESKD. For example, large population-based studies across multiple international cohorts have shown a mortality peak in the initial months following dialysis initiation.^15–17^ Furthermore, incident patients with ESKD transitioning to dialysis may exhibit frequent hospitalizations and readmissions, lower HRQOL, loss of independence, decline in physical function, increased symptom burden, and higher healthcare costs.^3,4,18^ Furthermore, large multicenter trial data did not show that planned early initiation of dialysis conferred improved survival nor clinical outcomes as compared with late-start dialysis.^19,20^ Recognizing these limitations of dialysis therapy, the federally mandated Advancing American Kidney Health Initiative unveiled in 2019 has recommended the adoption of treatments that slow or stop CKD progression,^21,22^ which has elevated the role of conservative and preservative management to the forefront of clinical care.

## Concept of Conservative and Preservative Management

**Figure 1.**
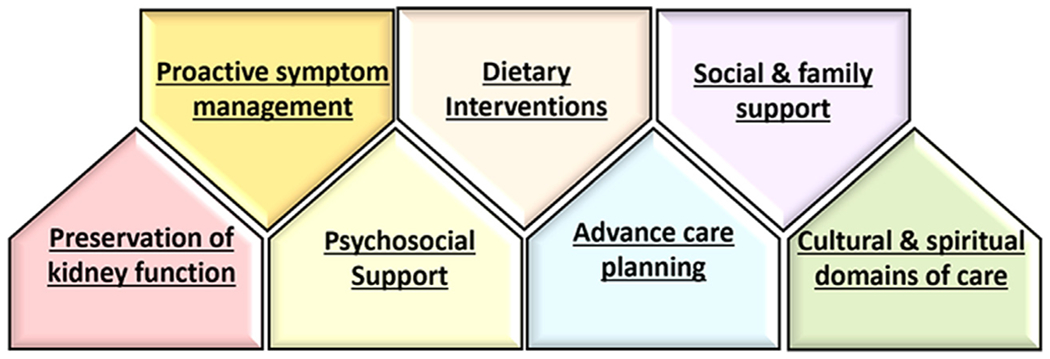
Multifaceted approach in the conservative and preservative management of kidney disease.

The concept of conservative and preservative management is centered on providing 1) active and comprehensive medical management of advanced CKD using nondialytic strategies and 2) ameliorating CKD progression, with the primary objectives of a) optimizing HRQOL, b) treating symptoms and complications of ESKD without dialysis or kidney transplantation, and c) preserving residual kidney function.^3,4^ It should also be clarified that conservative and preservative care do not equate to “no care” or “rationing of care,” and in fact require a multifaceted and holistic approach in which dietary interventions are a major cornerstone in management (Figure 1). Below we discuss the rationale for specific dietary interventions in conservative and preservative care, including 1) low dietary protein intake, largely from plant-dominant protein sources, 2) higher fruit and vegetable consumption, 3) increased dietary fiber intake, and 4) maintaining adequate caloric intake.

## Low-Protein Diets in Conservative and Preservative Management

### Clinical Practice Guidelines and Scientific Premise for Low-Protein Diets

In 2020, the National Kidney Foundation and Academy of Nutrition and Dietetics with endorsement from the International Society of Renal Nutrition and Metabolism (ISRNM) released an update to the Kidney Disease Outcomes Quality Initiative (KDOQI) Clinical Practice Guidelines for Nutrition in CKD (Table 1).^23^ These highly anticipated guidelines provide precise recommendations for the amount of dietary protein intake among NDD patients with CKD following rigorous review of high-quality data, and have been emphasized by international commentaries highlighting areas for potential local adaption and corollary research.^24–27^ More specifically, the KDOQI guidelines advise that adults with stages 3 to 5 NDD-CKD without diabetes who are metabolically stable should consume 1) a low-protein diet of 0.55-0.60 g/kg body weight/day, or 2) a very-low-protein diet of 0.28-0.43 g/kg of body weight/day with additional keto acid analogs in order to reduce risk of ESKD and/or death and to improve HRQOL based on grade 1A and 2C evidence. Among adults with stages 3 to 5 NDD-CKD with diabetes, the KDOQI guidelines also indicate that it is reasonable to prescribe a lower dietary protein intake of 0.6 to 0.8 g/kg of body weight/day to maintain stable nutritional status and optimize glycemic control based on opinion-level evidence. However, it bears mention that the guidelines were based on randomized controlled trials (RCTs) only and did not include implementation studies. Furthermore, while the evidence on patients with NDD-CKD is scant, in spite of very few RCTs, large observational studies were not taken into consideration.

Although the ISRNM, the largest international nutritional organization for the kidney disease population, was not directly involved in formulating the updated 2020 KDOQI guidelines, multiple ISRNM members led and/or played prominent roles in the guideline development, and the ISRNM also convened a review panel of international experts evaluating the recommendations prior to public review.^28^ Following public release of the 2020 KDOQI guidelines, the ISRNM and international kidney nutrition leaders published a commentary following a review and critical analysis in support of the updated recommendations.^28^ The ISRNM commentary included one notable distinction from the updated KDOQI guidelines. ISRNM experts highlighted that it is reasonable for clinicians to aim for the lower end of a streamlined target of 0.6 to 0.8 g/kg/day regardless of CKD etiology, considering that 1) lower dietary protein targets (i.e., 0.28-0.43 g/kg/day) may be challenging to achieve, particularly in geographic catchments without access to keto acid analogs and/or kidney dietitians, and 2) in the low dietary protein trials, actual dietary protein intake was generally above 0.6 g/kg/day despite a prescribed target of 0.55 to 0.6 g/kg/day (Table 2).

It bears mention that large population-based data show a high intake of dietary protein among adults across all levels of kidney function. In a cross-sectional analysis of 16,872 adults from that National Health and Nutrition Examination Survey (NHANES) who underwent dietary protein intake assessment by 24-hour dietary recall, mean dietary protein intake among both participants with and without kidney dysfunction was higher than recommended guidelines for NDD-CKD by KDOQI and for healthy adults by the Institute of Medicine, respectively.^29^ These trends are not surprising given the popularity of various high-protein diets in secular culture as a means of countering obesity and dysglycemia^30^ and/or marketing trends to enhance products with added protein. However, in the setting of NDD-CKD, higher dietary protein intake has ill effects on kidney function and structure vis-à-vis dilation of the glomerular afferent arterioles, leading to glomerular hyperfiltration, increased intraglomerular pressure, and damage to the glomerular structure.^30–32^ In contrast, lower dietary protein intake leads to afferent arteriole vasoconstriction, thereby reducing intraglomerular pressure, glomerular hyperfiltration, and proteinuria.^33–35^

### Data on the Efficacy/Effectiveness and Safety of Low-Protein Diets

A large body of evidence has shown that lower dietary protein intake is associated with decreased proteinuria, CKD progression, and uremic complications.^36–40^ For example, in a meta-analysis of 16 RCTs of low dietary protein intake in NDD-CKD, pooled data showed that lower protein intake of <0.8 g/kg/day was associated with lower risk of ESKD and all-cause mortality. The meta-analysis also demonstrated improved biochemical parameters including higher serum bicarbonate levels, decreased serum phosphorus levels, and less azotemia, as compared with a dietary protein intake of ≥0.8 g/kg/day.^40^ Although the Modification of Diet in Renal Disease trial showed marginal reduction in CKD progression with a very-low-protein diet, reanalysis of both achieved and prescribed dietary protein intake showed that low-protein diets significantly reduced eGFR decline.^41–43^ Other clinical trials and observational studies have corroborated the benefits of low-protein diets on CKD outcomes^36–39^ and survival.^44^

The safety of low-protein diets in NDD-CKD has also been corroborated by clinical trial and observational data. In a meta-analysis of 7 RCTs of low-protein diets supplemented with keto acid analogs in NDD-CKD, compared with a normal protein diet, supplemented low-protein or very-low protein diets prevented eGFR decline without leading to differences in serum albumin, serum creatinine, nor other nutrition indices across different protein intake groups.^39^ A more recent meta-analysis of 17 NDD-CKD trials comparing low-protein diets supplemented with keto acid analogs versus regular diets or nonsupplemented low-protein diets showed that supplemented low-protein diets significantly preserved eGFR and reduced proteinuria, increased serum albumin, and demonstrated no differences in other nutritional indices (e.g., body mass index, skinfold thickness, mid-arm circumference, and lean body mass).^37^ The safety and efficacy of low-protein diets have also been observed in patients with NDD-CKD of older age.^35^ In a landmark RCT of older (>70 years) adults with advanced NDD-CKD (eGFR 5-7 mL/minute/1.73 m^2^) without diabetes in Italy who were randomized to a supplemented very-low protein vegan diet (comprised of dietary protein intake of 0.3 g/kg/day and keto acids, amino acids, and vitamins) versus dialysis without dietary protein intake restriction, those in the supplemented very-low protein vegan diet arm delayed dialysis by ~11 months, with both groups showing similar mortality rates and the dialysis group demonstrating higher rates of hospitalization.^36^

While many dietary regimens for CKD are inherently restrictive,^28^ as well as concerns about the potential risk of protein-energy wasting,^45–47^ these data suggest that low-protein diets can be safely administered in NDD-CKD, particularly when implemented under the supervision of specialty-trained kidney dietitians (see “Practical Implementation of Dietary Interventions” below).^23,28,48–50^ Furthermore, although there are misconceptions that elderly patients with CKD typically have spontaneous reduction in dietary protein intake thereby making low-protein diets (LPDs) futile in this context, clinical studies have shown that most elderly patients consume higher-than-recommended dietary protein intake^51^; hence, there is indeed need for personalized nutritional management in this subpopulation.^51,52^ Additionally, a consensus paper from the European Society for Clinical Nutrition and Metabolism and the European Renal Nutrition group of the European Renal Association has underscored that individual risk-benefit assessment and appropriate nutritional monitoring should guide clinical decision-making in elderly patients with CKD.^52^ There has also been growing emphasis on the avoidance of excessive restriction and the optimization of the palatability and overall quality of life with more patient-centered dietary approaches. For example, long-term follow-up data from the TOrina-Pisa study showed high dietary satisfaction and minimal dropout among stages 3 to 5 patients with NDD-CKD who underwent moderately restricted LPDs (0.6 g/kg/day).^53^ This was also corroborated in a study of 153 patients with stages 3 to 5 NDD-CKD from France and Italy who underwent a personalized approach toward protein restriction.^54^

## Plant-Dominant Diets in Conservative and Preservative Management

### Clinical Practice Guidelines and Scientific Premise

A growing body of evidence has also underscored that, in addition to the *amount* of dietary protein intake, dietary protein *source* also has an important bearing on clinical outcomes in the kidney disease population.^55–57^ While the 2020 KDOQI guidelines indicate insufficient evidence to recommend a particular type of protein (plant vs. animal), the updated recommendations do support prescribing greater fruit and vegetable intake in patients with stages 1 to 4 NDD-CKD in order to decrease body weight, blood pressure, and net acid production.^23^ Whereas the KDOQI guidelines relied heavily on RCT data to limit risk of bias, the ISRNM commentary advised that an increasing number of observational data and clinical trials published following the KDOQI guidelines support plant-based protein and/or dietary patterns that may be reasonable for clinicians to consider.^28^

### Evidence of Efficacy/Effectiveness

Multiple observational studies have demonstrated that plant-based protein sources are associated with decreased risk of incident and prevalent CKD, CKD progression, and mortality risk. In a cross-sectional analysis of 420 adults with type 2 diabetes from the Dutch DIAbetes and LifEstyle Cohort Twente-1 cohort, participants completed food frequency questionnaires (FFQs) of the amounts of vegetable versus animal protein intake. Among participants with a dietary protein consumption of <0.8 g/kg/day, those with greater intake of protein from vegetable sources (defined as the highest tertile of vegetable protein intake) had a lower likelihood of prevalent CKD.^58^ With respect to developing de novo CKD, a longitudinal study of 11,952 Atherosclerosis Risk in Communities study participants with normal baseline kidney function completed two FFQ assessments over a median follow-up of 23 years. Participants with greater consumption of red and processed meats had higher risk of incident CKD, whereas those with higher dietary intake of nuts, legumes, and low-fat dairy had lower risk of developing CKD.^59^ Longitudinal data from the Nurses Health Study has also shown that both protein amount and source have a bearing on CKD progression.^60^ In 1624 women who completed semiquantitative FFQ’s, among those who had mild kidney dysfunction (eGFR’s ≥ 55-<80 mL/minute/1.73 m^2^) followed over 11 years, both higher dietary protein intake and intake from nondairy animal protein sources (as opposed to vegetable and dairy protein sources) were each associated with greater decline in eGFR over time. In a more recent observational study of 27,604 participants from the NHANES who underwent 24-hour dietary recall, both higher dietary protein intake of ≥1.4 g/kg/day compared to lower intake of 0.6 to <1.0 g/kg/day and intake from high biological value sources (i.e., sources with an amino acid composition similar to human proteins, which are typically from animal sources) were each associated with higher mortality risk.^44^ Greater adherence to dietary patterns with more plant-based foods such as the Dietary Approaches to Stop Hypertension (DASH), Mediterranean vegetarian, and vegan diets have also been associated with lower risk of incident CKD and ESKD.^61–65^ Longitudinal data from a study of 449 patients with NDD-CKD from Italy who underwent moderately restricted LPDs have also shown that this dietary intervention leads to longer dialysis-free follow-up time with comparable survival to dialysis at a lower cost.^66^

In addition to the aforementioned Brunori et al. study,^36^ a growing number of clinical trials have also examined the effects of plant-based diets in NDD-CKD. An RCT of 41 patients with diabetic kidney disease examined the effect of a soy protein diet (comprised of 65% soy/vegetable proteins and 35% animal proteins) versus a control diet (comprised of 70% animal protein and 30% vegetable protein). Participants in the soy protein arm demonstrated a significant reduction in proteinuria, glucose, lipids, and inflammatory marker levels over a 4-year follow-up period.^67^ In another trial of nondiabetic NDD-CKD with eGFR <30 mL/minute/1.73 m^2^ without substantial proteinuria (<1 g/g urinary creatinine), 207 participants were randomized to a keto acid analog supplemented vegetarian very-low-protein diet (0.3 g/kg/day of vegetable proteins) versus a conventional low-protein diet (0.6 g/kg/day). The study demonstrated that the supplemented vegetarian very-low protein diet was nutritionally safe and led to deferral of dialysis initiation.^68^

### Not All Plant-Based Diets are Low-Protein Diets

While various plant-based or plant-dominant diets (Table 2) including the DASH and Mediterranean diets confer a number of salutary benefits, it is important to highlight that they are not all per se low-protein diets.^69^ In a cross-sectional study of 71,851 participants in the Adventist Health-2 cohort who completed FFQs, those who were strict vegetarians had a similar amount of total protein intake compared those who were semi-, pesco-, lactoovo, and non-vegetarians.^70^ Additionally, strict vegetarians who were consumers of the highest amount of plant-based foods exceeded their minimum requirements for dietary protein intake. Similarly, data from the European Prospective Investigation into Cancer and Nutrition-Oxford study have shown that participants who consumed plant-based diets averaged ~13% of energy intake from protein, exceeding the minimum recommendations from United Kingdom and United States guidelines.^71,72^

Given strong evidence supporting the role of low-protein diets in delaying CKD progression, experts in the field have proposed a patient-centered, pragmatic plant-dominant low-protein diet (PLADO) comprised of 1) dietary protein intake of 0.6 to 0.8 g/kg/day from 50% plant-based sources, 2) higher fiber intake of >25 g/day, 3) low-sodium intake of <4 g/day (<3 g/day if edema or hypertension are present), and 4) adequate caloric intake of 30 to 35 kcal/kg/day administered by dietitians trained in NDD-CKD care.^57,73,74^ There are currently ongoing studies and trials evaluating the efficacy and safety of the PLADO diet in patients with NDD-CKD, as well as its counterpart in diabetic kidney disease (known as the plant-focused low-protein diet for chronic kidney disease in diabetes “PLAFOND” diet) (Table 3).^75,76^ Mixed methods research has also investigated the potential challenges as well as approaches to optimize successful implementation of plant-based diets by kidney dietitians and other multidisciplinary team members.^77^

## Salutary Impact of Plant-Based Diets on Uremic Complications

**Figure 2.**
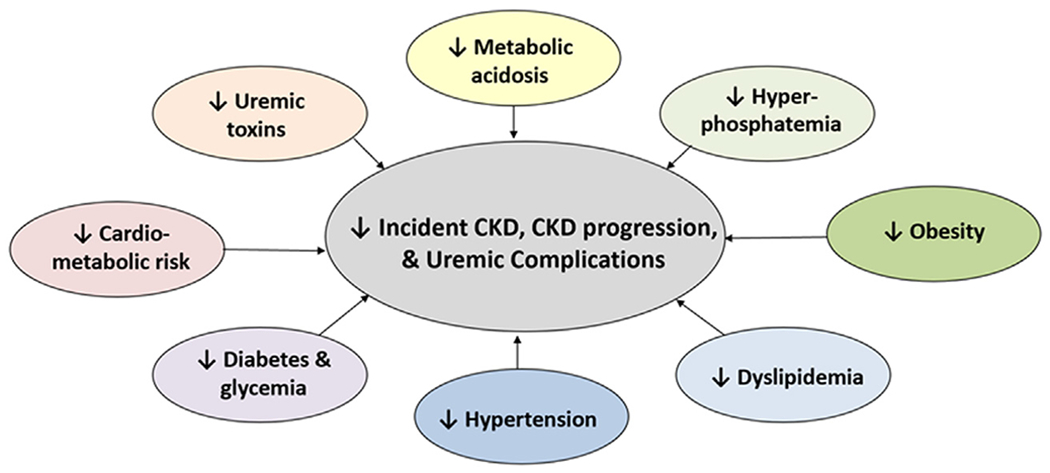
Benefits of a PLADO in the conservative and preservative management of CKD. CKD, chronic kidney disease.

In addition to delaying or averting the need for renal replacement therapy by decreasing CKD progression, a PLADO diet may have salutary effects on other uremic complications, including reduction of metabolic acidosis, mineral and bone disorders, and uremic toxin generation (Figure 2).^57,73,74^

### Metabolic Acidosis

Whereas animal-based proteins increase dietary acid load in CKD, plant-based foods are acid-neutral or alkali-producing.^56,78^ Acid is generated from animal protein intake due to the oxidization of the amino acids methionine and cysteine to inorganic sulfate.^56^ In contrast, plant-based foods contain citrate and malate as a form of natural dietary alkali, which is then converted to bicarbonate. On average, the Western diet (high in animal protein and low in plant-based protein) generates a dietary acid load of ~1 mEq/kg/day (i.e., 70 mEq/day in a 70-kg person). Decreased net endogenous acid production may in turn lead to decreased endothelin I, aldosterone, and angiotensin II levels and preservation of glomerular filtration rate, as well as prevention of metabolic acidosis leading to decreased bone resorption, insulin resistance, and sarcopenia.^79^

Based on the premise that acidosis increases kidney angiotensin II, which in turn mediates CKD progression in experimental models,^56,79^ well-designed clinical studies have sought to test the hypothesis that fruits and vegetables may be effective in reducing metabolic acidosis, kidney angiotensin II, and eGFR decline.^80,81^ In a recent clinical trial of stage 3 NDD-CKD and normal plasma total CO_2_ levels (22-24 mmol), 108 patients were randomized to usual care versus interventions to reduce dietary acid by 50% using sodium bicarbonate or fruits and vegetables. Participants in the usual care arm experienced a reduction in plasma total CO_2_ and increased urinary angiotensinogen (i.e., proxy of kidney angiotensin II) levels, whereas those in the bicarbonate or fruits and vegetables arm experienced higher total CO_2_ and decreased urinary angiotensinogen levels over time.^81^ Although creatinine- and cystatin C–based eGFR levels decreased in both arms, there was lesser decline in the intervention versus usual care arm after 3 years of follow-up. Another clinical trial of 71 patients with stage 4 NDD-CKD due to hypertension with low plasma total CO_2_ levels (<22 mmol) randomized participants to daily oral sodium bicarbonate therapy versus fruits and vegetables dosed to reduce dietary acid by 50% over 1 year. Participants in the fruits and vegetables arm achieved higher plasma total CO_2_ levels consistent with improved metabolic acidosis, as compared with the sodium bicarbonate arm; both groups experienced improved urine indices of kidney injury without increases in plasma potassium levels after 1 year.^80^ These data suggest that a diet emphasizing fruits and vegetables may be a preferable alternative (and perhaps more palatable option without added sodium load) than alkali supplements as a means to treat metabolic acidosis.^78^

### Mineral and Bone Disorders

The bulk of the body’s exogenous phosphorus load is from dietary intake, including organic and inorganic sources.^82–84^ While inorganic sources (e.g., food additives) more predominantly contribute to hyperphosphatemia due to their high bioaccessibility (i.e., as high as 100%), organic sources such as dietary protein are also a key contributor to the dietary phosphate load. Notably, phosphorus from animal proteins (which occurs in the form of caseins for dairy products) have a higher bioaccessibility (40%-60%) than phosphorus from plant-based proteins (which are often found in phytates) which have a lower bioaccessibility of 20% to 40% due to the inability of the small intestine to degrade phytate as it does not express phytase.^85^ In a report of data from NHANES, Moore et al. demonstrated that the inorganic phosphates added to the food supply are associated with small but significant increases in serum phosphorus even when controlling for levels of kidney function.^86^

Clinical studies have also corroborated that plant versus animal protein sources influence phosphorus homeostasis in CKD. In a crossover trial of nine patients with NDD-CKD who underwent 7 days of a vegetarian diet and 7 days of a meat diet with equivalent nutrients, receipt of the vegetarian diet led to lower serum phosphorus and fibroblast growth factor-23 levels, as well as decreased phosphaturia as compared with the meat diet.^87^ Recognizing the limitations of traditional methods of assessing dietary phosphate load, kidney nutrition investigators have developed a novel “Phosphatemic Index” to evaluate phosphate load based on its bioavailability.^88^ Recently, 20 healthy adults were administrated ten different test foods each containing 200 mg of phosphorus in order to assess their Phosphatemic Index. The Phosphatemic Index was calculated from the area under the curve of time versus serum phosphorus concentration curves. The study demonstrated that foods from animal sources (e.g., milk/dairy, pork/ham, etc.) tended to have higher Phosphatemic Index values versus foods from plant-based sources (e.g., soy/tofu). Subsequent ingestion of high Phosphatemic Index test meals led to higher serum fibroblast growth factor-23 levels and lower activated vitamin D levels compared with ingestion of lower Phosphatemic Index test meals.^88^

### Uremic Toxin Generation

Growing data also show that a plant-dominant diet reduces the generation of uremic toxins, likely due to a greater content of fiber.^57,73,74^ Dietary fiber, a nondigestible form of carbohydrate found in plant-based foods such as fruits, vegetables, whole grains, legumes, pulses, nuts, and seeds, and has been associated with a number of other health benefits, including improvement in blood pressure, glycemic control, dyslipidemia, weight modulation, constipation, and intestinal microbiota composition and function.^89^ Moreover, fiber intake correlates with a decrease in the inflammatory state, delays the progression of renal disease, and reduces overall mortality in nephropathic patients.^90,91^ In addition to a plant-dominant, fiber-rich diet leading to a reduction in gut bacteria-derived toxins such as trimethylamine N-oxide (i.e., a risk factor for insulin resistance and cardiovascular disease), greater intake of fiber may increase the generation of favorable short-chain fatty acids that may improve insulin sensitivity and reduce systemic inflammation.^92^ In a study of 15 vegetarians and 11 individuals who consumed an unrestricted diet with normal kidney function and underwent measurement of the urinary excretion of two solutes generated by colon bacteremia and found in uremia, namely p-cresol sulfate (PCS) and indoxyl sulfate (IS), both PCS and IS excretion were observed to be lower in vegetarians.^93^ Additionally, among vegetarians, lower excretion of PCS and IS were observed with higher fiber and lower protein intake. In a clinical trial of 56 maintenance hemodialysis patients who were randomized to daily supplements containing resistance starch (i.e., a form of dietary fiber) versus control starch for 6 weeks, those in the resistance starch arm experienced significant reductions in unbound/free IS and PCS levels and total IS levels, although the reduction in total PCS levels were not significant as compared with the control arm.^94^

## Practical Implementation of Dietary Interventions

The effective and safe implementation of nutritional advice in the conservative and preservative management of NDD-CKD require the involvement of and collaboration with specialty-trained kidney dietitians and/or international equivalents.^48–50^ Both the ISRNM and KDOQI recommendations underscore the essential role that kidney dietitians play in individualized medical nutritional therapy, as well as routine nutritional assessments.^23,28^ A patient-centered approach involves a shared understanding of treatment goals, effective communication to alleviate anxieties around food or food misconceptions, individualized advice, and assistance with implementation of dietetic advice in the face of a large symptom burden. In addition, attention to the dietary pattern rather than individual foods is important, and consideration given to the important cultural and social roles of food. Recognizing that some geographic catchments (i.e., developing countries) and/or health-care systems may have limited availability of and/or inadequate infrastructure to support the involvement of kidney dietitians, expanded use of telehealth and telemedicine services may be options to circumvent these barriers in access.^28^ Additionally, the ISRNM and other organizations such as the National Kidney Foundation and Academy of Nutrition and Dietetics have pioneered various in-person and tele-learning platforms to support the training of dietitians and other clinicians in kidney nutrition.^95,96^

Several innovative clinical models and studies have shown that providing patients with more personalized options (“multiple choice system”) and flexibility promotes adherence, dietary satisfaction, and quality of life. In a feasibility study of 131 patients with advanced CKD who were offered three dietary options, namely normalization of protein intake (0.8 g/kg/day), moderate protein restriction (0.6 g/kg/day) with a traditional mixed protein diet, or moderate protein restriction with a plant-based diet supplemented with keto acids, using an individualized stepwise approach, protein restriction was found to be feasible and was associated with stable nutritional status in a population largely of elderly age and high comorbidity burden.^51^ In another study of patients with severe or rapidly progressive NDD-CKD who were offered three LPD options (i.e., vegan diets supplemented with alpha-keto acids and essential amino acids; protein-free food in substitution of normal bread and pasta; or other diet, such as traditional, vegan nonsupplemented and tailored), excellent adherence was observed across all LPD arms without observed differences in mortality or timing of dialysis initiation.^66^

Another key consideration in the practical implementation of these dietary interventions is their pairing with evidence-based pharmacotherapies. As one example, while the traditional paradigm has been to restrict dietary potassium intake in moderate-to-advanced CKD given concerns about the ill effects of hyperkalemia, there is lack of evidence to support the benefits of this practice,^89,97^ and limited observational data have shown weak associations between dietary potassium intake and serum potassium levels.^98^ Furthermore, restricting heart-healthy fruits and vegetables and dietary patterns with a greater potassium content (i.e., DASH, PLADO/plant-focused low-protein diet for chronic kidney disease in diabetes diets) could potentially have negative effects on the cardiovascular health and survival of patients with CKD.^57,89,97^ In a retrospective analysis of 3,172 NHANES participants with kidney dysfunction who underwent dietary potassium and fiber intake assessment by 24-hour dietary recall, those who had concomitant low potassium and fiber intake had higher death risk versus those with high potassium and fiber consumption.^89^ Similarly, in a multicenter prospective observational study of 415 hemodialysis patients who underwent protocolized FFQs, lower dietary potassium intake was associated with worse survival.^97^ Hence, the utilization of novel potassium binders (if available) may allow for liberalization of dietary potassium intake to support greater consumption of PLADO diets, as well as continuing reno-protective renin-angiotensin-aldosterone inhibitors and nonselective steroidal mineralocorticoid receptor antagonists and avoiding/delaying the need for renal replacement therapy. Additionally, low-protein diets may have synergistic effects when used in conjunction with renin-angiotensin-aldosterone inhibitor and novel sodium-glucose cotransporter-2 inhibitors in attenuating proteinuria and CKD progression.^99^

## Conclusion

In summary, while dialysis has been the dominant treatment paradigm in patients with advanced kidney disease who are ineligible for or unlikely to receive kidney transplantation, growing interest in conservative and preservative care of NDD-CKD has emerged and dietary interventions are central to management. While solid evidence supports the use of low-protein diets in ameliorating CKD progression, further research including clinical trials and rigorous longitudinal observational studies are needed to determine the impact of PLADOs on kidney health outcomes and patient-centered endpoints, including HRQOL, functional status, and symptom burden. Furthermore, using an individualized approach that incorporates multidisciplinary collaboration with specialty-trained kidney dietitians can enhance the effectiveness, safety, and adherence to dietary interventions in the conservative and preservative care of CKD. Finally, an optimal approach in the nutritional management of CKD should emphasize shared decision-making with patients and their care partners, education and counseling regarding evidence-based dietary approaches, personalized nutritional advice, and effective communication and discussion of shared goals that includes consideration of patient-centered outcomes, including HRQOL and alleviation of symptom burden.

## Practical Application

Diet plays an important role in the conservative management and preservation of kidney function. Guidelines recommend low-protein diets to reduce the progression of kidney disease. There is also increasing research showing that plant-based low-protein diets may benefit kidney health.

## Figures and Tables

**Table 1.** Clinical Practice Guidelines on Dietary Protein Intake in Adult Patients With NDD-CKD

	Kidney Disease Outcomes Quality Initiative	International Society of Renal Nutrition and Metabolism
NDD-CKD without diabetes	Among patients with stage 3-5 NDD-CKD who are metabolically stable, recommend	Reasonable for clinicians to aim for the lower end of a streamlined target of 0.6-0.8 g/kg/d, regardless of CKD etiology.
	• Low protein diet of 0.55-0.6 g/kg/d, or • Very low protein diet of 0.28-0.43 g/kg/d + additional keto acids to meet protein requirements (0.55-0.60 g/kg/d)	• Lower dietary protein targets may be challenging to achieve without compromising energy intake or in settings without access to ketoanalog supplementation.
NDD-CKD with diabetes	Among patients with stage 3-5 NDD-CKD who are metabolically stable, recommend low protein diet of 0.6-0.8 g/kg/d.	• In low-protein trials, actual dietary protein intake was generally above 0.6 g/kg/d despite a prescribed target of 0.55-0.6 g/kg/d.

CKD, chronic kidney disease; NDD, nondialysis dependent.

**Table 2.** Various Types of Plant-Based Dietary Regimens

Plant-Based Diet	Description
PLADO diet	Plant-dominant low-protein diet for chronic kidney disease: 0.6-0.8 g/kg per d of dietary protein with at least 50% from plant-based sources, dietary sodium <4 g/d and dietary energy of 30-35 kcal per kilogram of ideal body weight per d.
PLAFOND diet	Plant-focused low-protein diet for chronic kidney disease in diabetes: 0.6-0.8 g/kg per d of dietary protein with at least 50% from plant-based sources, dietary sodium <4 g/d and dietary energy of 30-35 kcal per kilogram of ideal body weight per d.
DASH diet	Dietary strategy designed to reduce blood pressure characterized by a high intake of plant foods, in addition to reduced sodium and high calcium intake.
Mediterranean diet	Emphasizes whole plant foods from that geographical area with moderate consumption of lean meats, dairy, and seafood. Added sugars, processed foods, and red meat are generally excluded but healthy fats such as olive oil are included.
Flexiterian diet	Commonly referred to as a “semi-vegetarian.” Represents a diet that emphasizes plant-based foods but may periodically include meat and other animal-based foods.
Vegetarian diet	Excludes meat (beef, pork, chicken) but may include fish, dairy, or eggs and often specified as a pescatarian, lacto-vegetarian, or ovo-vegetarian, respectively.
Whole-food plant based diet	Emphasizes consumption of whole plant-based foods as opposed to refined or processed plant foods while still typically limiting animal-based foods.
Vegan diet	A diet and in some cases a lifestyle that avoids the use of products derived from animals.

DASH, dietary approaches to stop hypertension; PLADO, plant-dominant low-protein diet; PLAFOND, plant-focused low-protein diet for chronic kidney disease in diabetes.

**Table 3.** Comparison of the Traditional Diabetes and Kidney Diets to PLADO and PLAFOND Diets^57,76,100,101^

	Standard ADA Diet	Traditional (Nondialysis) Kidney Diet	PLADO and PLAFOND diets
Proportion of plant-based protein	25%-35%	<50%	50%-<75%
Total protein (g/kg/d)	0.8-1.4	0.6 to <0.8	0.6 to <0.8
Total protein (g/d)	96-112	0.6 to <0.8	0.6 to <0.8
Proportion of energy from protein	16%-19%	15%	8%-11%
Total plant-based protein (g/d)	24-34	<24-32	24-48
Total animal-based protein (g/d)	68-83	>24-32	12-32
Sodium intake (g/d)	<2.3	<2.3	<4*
Potassium intake (g/d)	3-5	<2	4.7

ADA, American Diabetes Association; PLADO, plant-dominant low-protein diet; PLAFOND, plant-focused low-protein diet for chronic kidney disease in diabetes.

* Lower sodium target of <3 g/d if edema or uncontrolled hypertension present.

## References

[1] Bolasco P, Kalantar-Zadeh K, Rhee C. Conservative management of chronic kidney disease: how to avoid or defer dialysis. Panminerva Med. 2017;59:115. 28290185 10.23736/S0031-0808.17.03298-0

[2] Rhee CM, Edwards D, Ahdoot RS, Living well with kidney disease and effective symptom management: consensus conference Proceedings. Kidney Int Rep. 2022;7:1951–1963. 36090498 10.1016/j.ekir.2022.06.015 PMC9459054

[3] Rhee CM, Nguyen DV, Nyamathi A, Kalantar-Zadeh K. Conservative vs. preservative management of chronic kidney disease: similarities and distinctions. Curr Opin Nephrol Hypertens. 2020;29:92–102. 31743240 10.1097/MNH.0000000000000573

[4] Zarantonello D, Rhee CM, Kalantar-Zadeh K, Brunori G. Novel conservative management of chronic kidney disease via dialysis-free interventions. Curr Opin Nephrol Hypertens. 2021;30:97–107. 33186220 10.1097/MNH.0000000000000670

[5] Davison SN, Levin A, Moss AH, Executive summary of the KDIGO Controversies conference on supportive care in chronic kidney disease: developing a roadmap to improving quality care. Kidney Int. 2015;88:447–459. 25923985 10.1038/ki.2015.110

[6] Bello AK, Okpechi IG, Osman MA, Epidemiology of peritoneal dialysis outcomes. Nat Rev Nephrol. 2022;18:779–793. 36114414 10.1038/s41581-022-00623-7 PMC9483482

[7] United States Renal Data System. 2022 USRDS Annual Data Report: Epidemiology of kidney disease in the United States. Bethesda, MD: National Institutes of Health, National Institute of Diabetes and Digestive and Kidney Diseases; 2022.

[8] Ko GJ, Obi Y, Chang TI, Factors associated with withdrawal from dialysis therapy in incident hemodialysis patients aged 80 years or older. J Am Med Dir Assoc. 2019;20:743–750.e1. 30692035 10.1016/j.jamda.2018.11.030 PMC6538473

[9] Ko GJ, Obi Y, Soohoo M, No survival benefit in octogenarians and Nonagenarians with extended hemodialysis treatment time. Am J Nephrol. 2018;48:389–398. 30423584 10.1159/000494336 PMC6583774

[10] “Transition in Care in Chronic Kidney Disease.” United States Renal Data System. In: 2017 USRDS Annual Data Report: Epidemiology of kidney disease in the United States. Bethesda, MD: National Institutes of Health, National Institute of Diabetes and Digestive and Kidney Diseases; 2017.

[11] Kalantar-Zadeh K, Lockwood MB, Rhee CM, Patient-centred approaches for the management of unpleasant symptoms in kidney disease. Nat Rev Nephrol. 2022;18:185–198. 34980890 10.1038/s41581-021-00518-z

[12] You A, Kalantar S, Norris K, Dialysis symptom index burden and symptom clusters in a prospective cohort of dialysis patients. J Nephrol. 2022;35:427–1436. 10.1007/s40620-022-01313-0 PMC9217843 35429297

[13] Mitch WE, Remuzzi G. Diets for patients with chronic kidney disease, still worth prescribing. J Am Soc Nephrol. 2004;15:234–237. 14694178 10.1097/01.asn.0000106014.20274.c7

[14] Eggers PW. Medicare’s end stage renal disease program. Health Care Financ Rev. 2000;22:55–60. 25372768 PMC4194691

[15] Foley RN, Chen SC, Solid CA, Gilbertson DT, Collins AJ. Early mortality in patients starting dialysis appears to go unregistered. Kidney Int. 2014;86:392–398. 24522495 10.1038/ki.2014.15

[16] Lukowsky LR, Kheifets L, Arah OA, Nissenson AR, Kalantar-Zadeh K. Patterns and predictors of early mortality in incident hemodialysis patients: new insights. Am J Nephrol. 2012;35:548–558. 22677686 10.1159/000338673 PMC4587354

[17] Robinson BM, Zhang J, Morgenstern H, Worldwide, mortality risk is high soon after initiation of hemodialysis. Kidney Int. 2014;85:158–165. 23802192 10.1038/ki.2013.252 PMC3877739

[18] Kalantar-Zadeh K, Kovesdy CP, Streja E, Transition of care from pre-dialysis prelude to renal replacement therapy: the blueprints of emerging research in advanced chronic kidney disease. Nephrol Dial Transplant. 2017;32(suppl_2):ii91–ii98. 28201698 10.1093/ndt/gfw357 PMC5837675

[19] Nesrallah GE, Mustafa RA, Clark WF, Canadian society of nephrology 2014 clinical practice guideline for timing the initiation of chronic dialysis. CMAJ (Can Med Assoc J). 2014;186:112–117. 24492525 10.1503/cmaj.130363 PMC3903737

[20] Cooper BA, Branley P, Bulfone L, A randomized, controlled trial of early versus late initiation of dialysis. N Engl J Med. 2010;363:609–619. 20581422 10.1056/NEJMoa1000552

[21] Mehrotra R. Advancing American kidney health: an introduction. Clin J Am Soc Nephrol. 2019;14:1788. 31690694 10.2215/CJN.11840919 PMC6895493

[22] Scott N. July 10, 2019, A day to be remembered: a patient perspective. Clin J Am Soc Nephrol. 2019;14:1798. 31694862 10.2215/CJN.10440919 PMC6895475

[23] Ikizler TA, Burrowes JD, Byham-Gray LD, KDOQI clinical practice guideline for nutrition in CKD: 2020 update. Am J Kidney Dis. 2020;76(3 Suppl 1):S1–S107. 32829751 10.1053/j.ajkd.2020.05.006

[24] Ikizler TA, Cuppari L. The 2020 updated KDOQI clinical practice guidelines for nutrition in chronic kidney disease. Blood Purif. 2021;50:667–671. 33652433 10.1159/000513698

[25] Cheng GP, Shi YY, Liu J, Zeng XQ, Liu Y. [Recommended intakes of protein and energy in patients with chronic kidney disease: from guidelines of KDOQI and KDIGO in 2020]. Zhonghua Yixue Zazhi. 2021;101:1287–1290. 34015869 10.3760/cma.j.cn112137-20201117-03117

[26] Naber T, Purohit S. Chronic kidney disease: role of diet for a reduction in the Severity of the disease. Nutrients. 2021;13:3277. 34579153 10.3390/nu13093277 PMC8467342

[27] Lambert K, Bahceci S, Harrison H, Commentary on the 2020 update of the KDOQI clinical practice guideline for nutrition in chronic kidney disease. Nephrology. 2022;27:537–540. 35118773 10.1111/nep.14025 PMC9303594

[28] Kistler BM, Moore LW, Benner D, The international Society of renal nutrition and metabolism commentary on the national kidney foundation and academy of nutrition and dietetics KDOQI clinical practice guideline for nutrition in chronic kidney disease. J Ren Nutr. 2021;31:116–120.e1. 32737016 10.1053/j.jrn.2020.05.002 PMC8045140

[29] Moore LW, Byham-Gray LD, Scott Parrott J, The mean dietary protein intake at different stages of chronic kidney disease is higher than current guidelines. Kidney Int. 2013;83:724–732. 23302719 10.1038/ki.2012.420

[30] Ko GJ, Rhee CM, Kalantar-Zadeh K, Joshi S. The effects of high-protein diets on kidney health and longevity. J Am Soc Nephrol. 2020;31:1667–1679. 32669325 10.1681/ASN.2020010028 PMC7460905

[31] Jhee JH, Kee YK, Park S, High-protein diet with renal hyperfiltration is associated with rapid decline rate of renal function: a community-based prospective cohort study. Nephrol Dial Transplant. 2020;35:98–106. 31172186 10.1093/ndt/gfz115

[32] Kalantar-Zadeh K, Fouque D. Nutritional management of chronic kidney disease. N Engl J Med. 2017;377:1765–1776. 29091561 10.1056/NEJMra1700312

[33] Kalantar-Zadeh K, Moore LW, Tortorici AR, North American experience with Low protein diet for Non-dialysis-dependent chronic kidney disease. BMC Nephrol. 2016;17:90. 27435088 10.1186/s12882-016-0304-9 PMC4952055

[34] Wang M, Chou J, Chang Y, The role of low protein diet in ameliorating proteinuria and deferring dialysis initiation: what is old and what is new. Panminerva Med. 2017;59:157–165. 27759735 10.23736/S0031-0808.16.03264-X

[35] Narasaki Y, Rhee CM, Kramer H, Kalantar-Zadeh K. Protein intake and renal function in older patients. Curr Opin Clin Nutr Metab Care. 2021;24:10–17. 33323714 10.1097/MCO.0000000000000712

[36] Brunori G, Viola BF, Parrinello G, Efficacy and safety of a very-low-protein diet when postponing dialysis in the elderly: a prospective randomized multicenter controlled study. Am J Kidney Dis. 2007;49:569–580. 17472838 10.1053/j.ajkd.2007.02.278

[37] Chewcharat A, Takkavatakarn K, Wongrattanagorn S, The effects of restricted protein diet supplemented with Ketoanalogue on renal function, blood pressure, nutritional status, and chronic kidney disease-mineral and bone disorder in chronic kidney disease patients: a systematic review and meta-analysis. J Ren Nutr. 2020;30:189–199. 31607548 10.1053/j.jrn.2019.07.005

[38] Fouque D, Laville M. Low protein diets for chronic kidney disease in non diabetic adults. Cochrane Database Syst Rev. 2009;(3):CD001892. 10.1002/14651858.CD001892.pub3 19588328

[39] Jiang Z, Zhang X, Yang L, Li Z, Qin W. Effect of restricted protein diet supplemented with keto analogues in chronic kidney disease: a systematic review and meta-analysis. Int Urol Nephrol. 2016;48:409–418. 26620578 10.1007/s11255-015-1170-2

[40] Rhee CM, Ahmadi SF, Kovesdy CP, Kalantar-Zadeh K. Low-protein diet for conservative management of chronic kidney disease: a systematic review and meta-analysis of controlled trials. J Cachexia Sarcopenia Muscle. 2018;9:235–245. 29094800 10.1002/jcsm.12264 PMC5879959

[41] Effects of dietary protein restriction on the progression of moderate renal disease in the modification of diet in renal disease study. J Am Soc Nephrol. 1996;7:2616–2626. 8989740 10.1681/ASN.V7122616

[42] Levey AS, Adler S, Caggiula AW Effects of dietary protein restriction on the progression ofadvanced renal disease in the modification ofdiet in renal disease study. Am J Kidney Dis. 1996;27:652–663. 8629624 10.1016/s0272-6386(96)90099-2

[43] Levey AS, Greene T, Beck GJ, Dietary protein restriction and the progression of chronic renal disease: what have all of the results of the MDRD study shown? Modification of diet in renal disease study group. J Am Soc Nephrol. 1999;10:2426–2439. 10541304 10.1681/ASN.V10112426

[44] Narasaki Y, Okuda Y, Moore LW, Dietary protein intake, kidney function, and survival in a nationally representative cohort. Am J Clin Nutr. 2021;114:303–313. 33742197 10.1093/ajcn/nqab011 PMC8246621

[45] Koppe L, Fouque D, Kalantar-Zadeh K. Kidney cachexia or protein-energy wasting in chronic kidney disease: facts and numbers. J Cachexia Sarcopenia Muscle. 2019;10:479–484. 30977979 10.1002/jcsm.12421 PMC6596400

[46] Ikizler TA, Cano NJ, Franch H, Prevention and treatment of protein energy wasting in chronic kidney disease patients: a consensus statement by the international society of renal nutrition and metabolism. Kidney Int. 2013;84:1096–1107. 23698226 10.1038/ki.2013.147

[47] Carrero JJ, Stenvinkel P, Cuppari L, Etiology of the protein-energy wasting syndrome in chronic kidney disease: a consensus statement from the International Society of Renal Nutrition and Metabolism (ISRNM). J Ren Nutr. 2013;23:77–90. 23428357 10.1053/j.jrn.2013.01.001

[48] Jimenez EY, Kelley K, Schofield M, Medical nutrition therapy access in CKD: a cross-sectional survey of patients and providers. Kidney Med. 2021;3:31–41.e1. 33604538 10.1016/j.xkme.2020.09.005 PMC7873758

[49] Kalantar-Zadeh K, Saville J, Moore LW. Unleashing the Power of renal nutrition in value-based models of kidney care choices: Leveraging dietitians’ Expertise and medical nutrition therapy to delay dialysis initiation. J Ren Nutr. 2022;32:367–370. 35589046 10.1053/j.jrn.2022.05.001

[50] Narasaki Y, Rhee CM. Dietary therapy for managing hyperphosphatemia. Clin J Am Soc Nephrol. 2020;16:9–11. 33380472 10.2215/CJN.18171120 PMC7792640

[51] Torreggiani M, Fois A, Moio MR, Spontaneously low protein intake in elderly CKD patients: Myth or Reality? Analysis of baseline protein intake in a large cohort of patients with advanced CKD. Nutrients. 2021;13:4371. 34959922 10.3390/nu13124371 PMC8707092

[52] Piccoli GB, Cederholm T, Avesani CM, Nutritional status and the risk of malnutrition in older adults with chronic kidney disease - implications for low protein intake and nutritional care: a critical review endorsed by ERN-ERA and ESPEN. Clin Nutr. 2023;42:443–457. 36857954 10.1016/j.clnu.2023.01.018

[53] Piccoli GB, Di Iorio BR, Chatrenet A, Dietary satisfaction and quality of life in chronic kidney disease patients on low-protein diets: a multicentre study with long-term outcome data (TOrino-Pisa study). Nephrol Dial Transplant. 2020;35:790–802. 31435654 10.1093/ndt/gfz147

[54] Fois A, Torreggiani M, Trabace T, Quality of life in CKD patients on low-protein diets in a multiple-choice diet system. Comparison between a French and an Italian experience. Nutrients. 2021;13:1354. 33919635 10.3390/nu13041354 PMC8073895

[55] Joshi S, Hashmi S, Shah S, Kalantar-Zadeh K. Plant-based diets for prevention and management of chronic kidney disease. Curr Opin Nephrol Hypertens. 2020;29:16–21. 31725014 10.1097/MNH.0000000000000574

[56] Joshi S, McMacken M, Kalantar-Zadeh K. Plant-based diets for kidney disease: a guide for clinicians. Am J Kidney Dis. 2021;77:287–296. 33075387 10.1053/j.ajkd.2020.10.003

[57] Kalantar-Zadeh K, Joshi S, Schlueter R, Plant-dominant low-protein diet for conservative management of chronic kidney disease. Nutrients. 2020;12:1931. 32610641 10.3390/nu12071931 PMC7400005

[58] Oosterwijk MM, Soedamah-Muthu SS, Geleijnse JM, High dietary intake of vegetable protein is associated with lower prevalence of renal function Impairment: results of the Dutch DIALECT-1 cohort. Kidney Int Rep. 2019;4:710–719. 31080926 10.1016/j.ekir.2019.02.009 PMC6506707

[59] Haring B, Selvin E, Liang M, Dietary protein sources and risk for incident chronic kidney disease: results from the Atherosclerosis risk in communities (ARIC) study. J Ren Nutr. 2017;27:233–242. 28065493 10.1053/j.jrn.2016.11.004 PMC5476496

[60] Knight EL, Stampfer MJ, Hankinson SE, Spiegelman D, Curhan GC. The impact of protein intake on renal function decline in women with normal renal function or mild renal insufficiency. Ann Intern Med. 2003;138:460–467. 12639078 10.7326/0003-4819-138-6-200303180-00009

[61] Banerjee T, Crews DC, Tuot DS, Poor accordance to a DASH dietary pattern is associated with higher risk of ESRD among adults with moderate chronic kidney disease and hypertension. Kidney Int. 2019;95:1433–1442. 30975440 10.1016/j.kint.2018.12.027 PMC6602537

[62] Hu EA, Steffen LM, Grams ME, Dietary patterns and risk of incident chronic kidney disease: the Atherosclerosis risk in communities study. Am J Clin Nutr. 2019;110:713–721. 31386145 10.1093/ajcn/nqz146 PMC6736122

[63] Liu HW, Tsai WH, Liu JS, Kuo KL. Association of vegetarian diet with chronic kidney disease. Nutrients. 2019;11:279. 30691237 10.3390/nu11020279 PMC6412429

[64] Mozaffari H, Ajabshir S, Alizadeh S. Dietary approaches to stop hypertension and risk of chronic kidney disease: a systematic review and meta-analysis of observational studies. Clin Nutr. 2020;39:2035–2044. 31669002 10.1016/j.clnu.2019.10.004

[65] Xu K, Cui X, Wang B, Tang Q, Cai J, Shen X. Healthy adult vegetarians have better renal function than matched omnivores: a cross-sectional study in China. BMC Nephrol. 2020;21:268. 32652943 10.1186/s12882-020-01918-2 PMC7353802

[66] Piccoli GB, Nazha M, Capizzi I, Diet as a system: an observational study investigating a multi-choice system of moderately restricted low-protein diets. BMC Nephrol. 2016;17:197. 27927186 10.1186/s12882-016-0413-5 PMC5142321

[67] Azadbakht L, Atabak S, Esmaillzadeh A. Soy protein intake, cardiorenal indices, and C-reactive protein in type 2 diabetes with nephropathy: a longitudinal randomized clinical trial. Diabetes Care. 2008;31:648–654. 18184902 10.2337/dc07-2065

[68] Garneata L, Stancu A, Dragomir D, Stefan G, Mircescu G. Ketoanalogue-supplemented vegetarian very low-protein diet and CKD progression. J Am Soc Nephrol. 2016;27:2164–2176. 26823552 10.1681/ASN.2015040369 PMC4926970

[69] Rhee CM, Narasaki Y “Effects of nutritional status and changes in nutrient intake on renal function”. In: Kopple, Massry, Kalantar-Zadeh, and Fouque’s Nutritional Management of Kidney Disease. 4th ed Elsevier; 2021.

[70] Rizzo NS, Jaceldo-Siegl K, Sabate J, Fraser GE. Nutrient profiles of vegetarian and nonvegetarian dietary patterns. J Acad Nutr Diet. 2013;113:1610–1619. 23988511 10.1016/j.jand.2013.06.349 PMC4081456

[71] Bradbury KE, Tong TYN, Key TJ. Dietary intake of high-protein foods and other major foods in meat-eaters, Poultry-eaters, Fish-eaters, vegetarians, and vegans in UK Biobank. Nutrients. 2017;9:1317. 29207491 10.3390/nu9121317 PMC5748767

[72] Davey GK, Spencer EA, Appleby PN, Allen NE, Knox KH, Key TJ. EPIC-Oxford: lifestyle characteristics and nutrient intakes in a cohort of 33 883 meat-eaters and 31 546 non meat-eaters in the UK. Public Health Nutr. 2003;6:259–269. 12740075 10.1079/PHN2002430

[73] Chen Y, Wu J, Yu D, Liu M. Plant or animal-based or PLADO diets: which should chronic kidney disease patients choose? J Ren Nutr. 2022;33:228–235. 35809890 10.1053/j.jrn.2022.06.011

[74] Sakaguchi Y, Kaimori JY, Isaka Y. Plant-dominant low protein diet: a potential alternative dietary practice for patients with chronic kidney disease. Nutrients. 2023;15:1002. 36839360 10.3390/nu15041002 PMC9964049

[75] “Plant-Focused nutrition in patients with diabetes and chronic kidney disease (PLAFOND)”. https://clinicaltrials.gov/ct2/show/NCT05514184. Accessed January 1, 2023.

[76] Kalantar-Zadeh K, Rhee CM, Joshi S, Brown-Tortorici A, Kramer HM. Medical nutrition therapy using plant-focused low-protein meal plans for management of chronic kidney disease in diabetes. Curr Opin Nephrol Hypertens. 2022;31:26–35. 34750331 10.1097/MNH.0000000000000761

[77] Stanford J, Zuck M, Stefoska-Needham A, Charlton K, Lambert K. Acceptability of plant-based diets for people with chronic kidney disease: perspectives of renal dietitians. Nutrients. 2022;14:216. 35011091 10.3390/nu14010216 PMC8747619

[78] Uribarri J, Oh MS. The key to halting progression of CKD might be in the produce market, not in the pharmacy. Kidney Int. 2012;81:7–9. 22170526 10.1038/ki.2011.331

[79] Carrero JJ, Gonzalez-Ortiz A, Avesani CM, Plant-based diets to manage the risks and complications of chronic kidney disease. Nat Rev Nephrol. 2020;16:525–542. 32528189 10.1038/s41581-020-0297-2

[80] Goraya N, Simoni J, Jo CH, Wesson DE. A comparison of treating metabolic acidosis in CKD stage 4 hypertensive kidney disease with fruits and vegetables or sodium bicarbonate. Clin J Am Soc Nephrol. 2013;8:371–381. 23393104 10.2215/CJN.02430312 PMC3586961

[81] Goraya N, Simoni J, Jo CH, Wesson DE. Treatment of metabolic acidosis in patients with stage 3 chronic kidney disease with fruits and vegetables or oral bicarbonate reduces urine angiotensinogen and preserves glomerular filtration rate. Kidney Int. 2014;86:1031–1038. 24694986 10.1038/ki.2014.83

[82] Brown-Tortorici AR, Narasaki Y, You AS, The Interplay between dietary phosphorous, protein intake, and mortality in a prospective hemodialysis cohort. Nutrients. 2022;14:3070. 35893923 10.3390/nu14153070 PMC9330827

[83] Kalantar-Zadeh K, Gutekunst L, Mehrotra R, Understanding sources of dietary phosphorus in the treatment of patients with chronic kidney disease. Clin J Am Soc Nephrol. 2010;5:519–530. 20093346 10.2215/CJN.06080809

[84] Kalantar-Zadeh K. Patient education for phosphorus management in chronic kidney disease. Patient Prefer Adherence. 2013;7:379–390. 23667310 10.2147/PPA.S43486 PMC3650565

[85] Ekramzadeh M, Moore LW, Kalantar-Zadeh K, Kopple JD. Phytate and kidney health: the roles of dietary phytate in inhibiting intestinal phosphorus absorption and Intravenous phytate in decreasing soft tissue calcification. J Ren Nutr. 2023;3:225–227. 10.1053/j.jrn.2023.01.001 36638857

[86] Moore LW, Nolte JV, Gaber AO, Suki WN. Association of dietary phosphate and serum phosphorus concentration by levels of kidney function. Am J Clin Nutr. 2015;102:444–453. 26040641 10.3945/ajcn.114.102715

[87] Moe SM, Zidehsarai MP, Chambers MA, Vegetarian compared with meat dietary protein source and phosphorus homeostasis in chronic kidney disease. Clin J Am Soc Nephrol. 2011;6:257–264. 21183586 10.2215/CJN.05040610 PMC3052214

[88] Narasaki Y, Yamasaki M, Matsuura S, Phosphatemic index is a novel evaluation Tool for dietary phosphorus load: a whole-foods approach. J Ren Nutr. 2020;30:493–502. 32778471 10.1053/j.jrn.2020.02.005

[89] Narasaki Y, You AS, Malik S, Dietary potassium intake, kidney function, and survival in a nationally representative cohort. Am J Clin Nutr. 2022;116:1123–1134. 36026516 10.1093/ajcn/nqac215 PMC9535513

[90] Lu L, Huang YF, Wang MQ, Dietary fiber intake is associated with chronic kidney disease (CKD) progression and cardiovascular risk, but not protein nutritional status, in adults with CKD. Asia Pac J Clin Nutr. 2017;26:598–605. 28582807 10.6133/apjcn.072016.08

[91] Krishnamurthy VM, Wei G, Baird BC, High dietary fiber intake is associated with decreased inflammation and all-cause mortality in patients with chronic kidney disease. Kidney Int. 2012;81:300–306. 22012132 10.1038/ki.2011.355 PMC4704855

[92] Lau WL, Tran T, Rhee CM, Kalantar-Zadeh K, Vaziri ND. Diabetes and the gut Microbiome. Semin Nephrol. 2021;41:104–113. 34140089 10.1016/j.semnephrol.2021.03.005

[93] Patel KP, Luo FJ, Plummer NS, Hostetter TH, Meyer TW. The production of p-cresol sulfate and indoxyl sulfate in vegetarians versus omnivores. Clin J Am Soc Nephrol. 2012;7:982–988. 22490877 10.2215/CJN.12491211 PMC3362314

[94] Sirich TL, Plummer NS, Gardner CD, Hostetter TH, Meyer TW. Effect of increasing dietary fiber on plasma levels of colon-derived solutes in hemodialysis patients. Clin J Am Soc Nephrol. 2014;9:1603–1610. 25147155 10.2215/CJN.00490114 PMC4152802

[95] Kopple JD, Karupaiah T, Chan M, Burrowes JD, Kirk J, Prest M. Global renal Internet Course for dietitians (GRID Course). J Ren Nutr. 2022;32:131–134. 33812799 10.1053/j.jrn.2021.02.005

[96] Kalantar-Zadeh K, Moore LW. Renal Telenutrition for kidney health: Leveraging telehealth and telemedicine for nutritional assessment and dietary management of patients with kidney disorders. J Ren Nutr. 2020;30:471–474. 33168145 10.1053/j.jrn.2020.09.003

[97] Narasaki Y, Okuda Y, Kalantar SS, Dietary potassium intake and mortality in a prospective hemodialysis cohort. J Ren Nutr. 2021;31:411–420. 33121888 10.1053/j.jrn.2020.05.008 PMC8614638

[98] Clase CM, Carrero JJ, Ellison DH, Potassium homeostasis and management of dyskalemia in kidney diseases: conclusions from a kidney disease: improving global outcomes (KDIGO) controversies conference. Kidney Int. 2020;97:42–61. 31706619 10.1016/j.kint.2019.09.018

[99] Kalantar-Zadeh K, Beddhu S, Kovesdy CP, Kramer HJ, Fouque D. Biologically plausible trends suggesting that a low-protein diet may enhance the effect of flozination caused by the sodium-glucose cotransporter-2 inhibitor dapagliflozin on albuminuria. Diabetes Obes Metab. 2021;23:2825–2826. 34387935 10.1111/dom.14524

[100] Cupisti A, Gallieni M, Avesani CM, D’Alessandro C, Carrero JJ, Piccoli GB. Medical nutritional therapy for patients with chronic kidney disease not on dialysis: the low protein diet as a Medication. J Clin Med. 2020;9:3644. 33198365 10.3390/jcm9113644 PMC7697617

[101] Ko GJ, Kalantar-Zadeh K, Goldstein-Fuchs J, Rhee CM. Dietary approaches in the management of diabetic patients with kidney disease. Nutrients. 2017;9:824. 28758978 10.3390/nu9080824 PMC5579617

